# A Stronger Rhizosphere Impact on the Fungal Communities Compared to the Bacterial Communities in Pecan Plantations

**DOI:** 10.3389/fmicb.2022.899801

**Published:** 2022-06-30

**Authors:** Junping Liu, Yujie Tang, Jiashu Bao, Hankun Wang, Fangren Peng, Pengpeng Tan, Guolin Chu, Shuai Liu

**Affiliations:** ^1^Co-Innovation Center for Sustainable Forestry in Southern China, College of Forestry, Nanjing Forestry University, Nanjing, China; ^2^College of Biology and the Environment, Nanjing Forestry University, Nanjing, China

**Keywords:** rhizosphere soil, bulk soil, relevance networks, PGPRs, mycorrhizal fungi, pecan plantation

## Abstract

Understanding microbial communities associated with bulk and rhizosphere soils will benefit the maintenance of forest health and productivity and the sustainable development of forest ecosystems. Based on MiSeq sequencing, we explored the differences between the bulk soil and the rhizosphere soil on bacterial and fungal communities of pecan plantation. Results suggested that rhizosphere-associated fungal rather than bacterial community structures differed from bulk soil, and rhizosphere soil had lower fungal diversity than bulk soil. *Actinobacteria* and *Cantharellales* were the bacterial and fungal biomarkers of the rhizosphere soil of pecan plantation, respectively. In addition, *Pleosporales*, which are mainly involved in saprophylaxis and plant pathogenic processes, was identified as one of the most important fungal biomarkers for the bulk soil, and the FunGuild predicted a higher relative abundance of pathogenic fungi in bulk soil compared to rhizosphere soil. The pH, ammonium nitrogen (${\text{NH}}_{4}^{+}$-N), nitrate nitrogen (${\text{NO}}_{3}^{-}$-N), and total carbon (TC) contents drove microbial community structure and composition. The bacterial network was simpler in the rhizosphere soil than in the bulk soil. However, fungi showed the opposite network pattern. Keystone species in bacterial and fungal networks were mostly involved in nutrient cycling and the C cycling, and were found to be enriched in the rhizosphere soil. Overall, in terms of bacterial and fungal communities, the rhizosphere soil behaves more healthily than the bulk soil and has a higher potential for nutrient cycling.

## Introduction

Soil health is closely linked to global human health (Oliver and Gregory, 2015). As one of the most active parts of the soil, microorganisms are important in plant development and stress resistance (Liu et al., 2020a). Moreover, soil microorganisms are recognized as the important implementers of biogeochemical cycling and are major contributors to maintaining soil quality, sustainable agroforestry, and ecosystem functions (Wang J. L. et al., 2021; Xu Y. et al., 2021). Therefore, understanding the compositions and functions of soil microbial communities is important in the sustainable management of farmlands, forests, and human health.

Microbial interactions and microbial–plant interactions influence changes in the soil environment and the growth of crops or forest trees (Latz et al., 2021; Zheng et al., 2021). In turn, a variety of biotic and abiotic factors mediate the compositions and diversities of soil microbial communities (Cui et al., 2019; Zheng et al., 2021). The host plant itself is the most critical determinant of microbial community structure (Lejon et al., 2005; Adair and Douglas, 2017). Nutrients and signaling molecules released from plant roots promote the selection of specific taxa and functions of microorganisms, making rhizosphere soil different from bulk soil (Prashar et al., 2013; Uroz et al., 2016), which is known as the rhizosphere effect hypothesis. Any toxic or beneficial substances produced by rhizosphere microorganisms directly affect plant growth and health (Prashar et al., 2013). Usually, root exudates are used as a carbon source by soil microorganisms and form a nutrient-rich niche (Semenov et al., 2020), and the unstable carbon input from the roots will affect the soil carbon turnover and nitrogen mineralization (Finzi et al., 2015). Therefore, the rhizosphere has long been considered to be a hotspot.

Different interactions between microorganisms include predation, competition for resources and space, and mutually beneficial symbiosis, which constitute different microbial communities (Middleton et al., 2021). Soil microbial communities are sensitive to changes in soil physical and chemical properties, such as soil moisture, total carbon content, pH, and available nutrition contents (Cui et al., 2019; Essel et al., 2019; Yu et al., 2021; Ding et al., 2022). Commonly, pH has been reported to be the key driver affecting bacterial and fungal communities (Lauber et al., 2009; Cui et al., 2019; Praeg et al., 2019; Lopes et al., 2021). Previous studies have documented that the rhizosphere soil differed in bacterial or fungal communities from the bulk soil due to the differences in the soil physical and chemical properties (Essel et al., 2019; Ren et al., 2021). It was reported that rhizosphere soil had more microbial biomass than bulk soil (Zhang C. et al., 2012; Wu et al., 2021; Kumar and Garkoti, 2022). However, the α-diversity values of soil bacteria and fungi were usually found to be lower in rhizosphere soil, compared to those observed in bulk soil (Fan et al., 2017; Zhang et al., 2018; Essel et al., 2019).

Determining the assembly process of microbial communities and species correlations is critical to understanding the mechanisms related to community diversity (Valyi et al., 2016). Network analyses provide a new perspective for studying the complex microbial community compositions and functional traits (Mendes et al., 2014). Microbial keystone species are microorganisms in the central network that play a key role in maintaining biodiversity and its structure, function, and stability, and whose disappearance or weakening would result in fundamental changes to the microbial community (Xiao et al., 2021; Yang et al., 2021). By defining the degree of specific interactions between taxa nodes in an association network, keystone species have been identified in many environments (Fan et al., 2018; Liu et al., 2020b; Liu C. et al., 2021; Shi et al., 2021). Keystone species usually have clearer ecological capabilities, such as disease resistance (Berg et al., 2017), and are involved in carbon or nitrogen cycles (Mendes et al., 2014; Wang J. L. et al., 2021). Hence, exploration of keystone species can determine the health of the microbial network and the quality of the soil, further providing a theoretical basis for the management of plantations, such as fertilization and disease control.

Pecan [*Carya illinoinensis* (Wangenh.) K. Koch], a broadleaved forest tree with high fruit value, belongs to the genus *Carya* and the family *Juglandaceae* and is native to the United States (Zhang et al., 2015). Besides the high economic value of the fruits, pecan trees have straight trunks, making their timber highly compliant for furniture, flooring, and landscaping. Current studies of pecan have focused on the molecular mechanisms of plant breeding (Liu et al., 2020c; Zhu et al., 2020) and sought the best management practices (Shang et al., 2020; Liu J. et al., 2021), but little attention has been given to the belowground ecosystem.

Reports of rhizosphere microorganisms were mostly concentrated on grassland and farmland ecosystems (Nan et al., 2020; Latz et al., 2021; Otero-Jiménez et al., 2021); however, there are relatively few studies on woody plants in forest ecosystems, particularly there is no sufficient information on pecan plantation. Soil bacteria and fungi are the two largest groups, and their community composition and abundance can characterize soil health and productivity level (Xu H. et al., 2021; Yang et al., 2021). Therefore, in the current study, based on high-throughput sequencing technology, we explored communities of bacteria and fungi in the rhizosphere soil and bulk soil of pecan plantations. We hypothesized that the bacterial and fungal diversities in rhizosphere soil were higher than those observed in bulk soil, while community compositions and ecology networks in rhizosphere soil were simpler than those found in bulk soil, and that bacterial and fungal community structures would be vitally affected by soil pH. The objectives of this study were as follows: (1) reveal the impact of the differences between the bulk soil and the rhizosphere soil on the diversities, community structures, and potential functions of microorganisms of pecan plantations, and (2) identify the bacterial and fungal biomarkers and keystone species in the bulk and rhizosphere soil of pecan plantations. These results would help to improve our comprehensive understanding of rhizosphere microorganisms in pecan plantations and thus can stimulate the development of better management strategies, such as nutrition and disease control, exploitation of beneficial microorganisms, etc., which has potential significance for improving forest productivity and health.

## Materials and Methods

### Study Site and Soil Sampling

The study area was located in Jiangsu Province (116°18′-121°57′E, 30°45′-35°20′N), one of the main planting areas for pecan plantations in China. The climate in this area was controlled by the East Asian monsoon, with an average annual temperature between 13.6 and 16.1°C, and annual precipitation ranging from 704 to 1,250 mm. Three sites in northern, central, and southern Jiangsu Province, at different latitudes, were selected as test sites. The soil types in the three areas were Halosols, Cambosols, and Argosols, respectively, according to the soil taxonomy of the FAO. To ensure representative sampling, we selected the pecan plantations with different stand ages (7-, 12-, and 35-year-old) and different varieties (“Pawnee,” “Mahan,” and “Jinhua”) as research objects. A nested experimental design was applied, and a total of 84 samples of rhizosphere soil and bulk soil were collected, and the bacterial and fungal communities were studied. The specific experimental design is shown in Supplementary Figure S1. The samples were collected in early October 2020 at a depth of 0–60 cm and within a radius of 0.5 m from the trunk of trees. The loosely bound soil was shaken off, and the tightly adhered soil that served as rhizosphere soil was brushed off and then collected. Bulk soil without roots was also collected within 5–10 cm away from the root zone. The soil samples were transported to the laboratory in a foam box with Drikold, sieved through a 2-mm sieve, and then stored at −80°C in a refrigerator.

### Soil Chemistry Analysis

Soil pH was measured by a pH meter (pH 700, Eutech, San Francisco, CA, USA) with a 1:2.5 fresh soil to water ratio. Fresh soil was extracted with 2 M KCl to obtain the contents of soil ammonium nitrogen (${\text{NH}}_{4}^{+}$-N) and nitrate nitrogen (${\text{NO}}_{3}^{-}$-N), which were determined by a UV spectrophotometer (Shimadzu UVmini-1240, Agilent Technologies, Kyoto, Japan). The indophenol blue colorimetric method was applied to determine the ${\text{NH}}_{4}^{+}$-N content, and the dual-wavelength colorimetric method (OD values at 225 and 275 nm) was applied to determine the ${\text{NO}}_{3}^{-}$-N content. Soil available phosphorous (AP) and available potassium (AK) contents were extracted with 0.5 M NaHCO_3_ and 1 M NH_4_OAc, respectively, and were determined using a UV spectrophotometer (Shimadzu UVmini-1240, Agilent Technologies, Kyoto, Japan) and flame photometer, respectively (BWB XP, BWB Technologies, Newbury, UK). The Dumas combustion method and elemental analyzer (2400 Series II CHNS/O, PerkinElmer, Waltham, MA, USA) were used to determine the content of total carbon (TC) and total nitrogen (TN). After HF-HCLO_4_-H_2_SO_4_ digestion, the Mo-Sb colorimetric method and flame spectrophotometry method were applied for the determination of total phosphorus (TP) and total potassium (TK) content, respectively, for which a UV spectrophotometer (Shimadzu UVmini-1240, Agilent Technologies, Kyoto, Japan) and flame photometer (BWB XP, BWB Technologies, Newbury, UK) were used, respectively.

### DNA Extraction, Amplification, MiSeq Sequencing, and Bioinformatic Analysis

Soil DNA was extracted from 0.5 g of soil by using the Fast DNA SPIN Kit (MP Biomedicals, CA, USA) following the manufacturer's instructions. The integrity of the DNA was assessed by 1% agarose gel electrophoresis, and a NanoDrop 2000 spectrophotometer (Thermo Scientific, Wilmington, USA) was used to measure the purity and concentration of the extracted DNA sample. The primer pairs for amplification of the bacterial 16S rRNA gene and fungal internal transcribed spacer (ITS) rRNA genes were 338F (5′-ACTCCTACGGGAGGCAGCA-3′) and 806R (5′-GGACTACHVGGGTWTCTAAT-3′), and ITS1F (5′-CTTGGTCATTTAGAGGAAGTAA-3′) and ITS2R (5′-GCTGCGTTCTTCATCGATGC-3′), respectively. The primers and specific reaction conditions and amplification procedures were performed according to the method referred by Yan et al. (2021). The purified PCR products were sequenced on an Illumina MiSeq PE300 platform (Illumina, San Diego, USA), by Shanghai Majorbio Biopharm Biotechnology Co., Ltd. (Shanghai, China). The sequences were uploaded to the NCBI Sequence Read Archive (SRA) under accession numbers PRJNA777007 (bacteria) and PRJNA777363 (fungi).

After quality control, filtering, and splicing by fastp (version 0.20.0) (Chen et al., 2018) and FLASH (version 1.2.7) (Magoč and Salzberg, 2011), the effective tags were obtained. The raw sequences were denoised, sorted, and then clustered into operational taxonomic units (OTUs) at 97% similarity by UPARSE (version 7.1) (Edgar, 2013). Taxonomic analysis of representative OTU sequences with 97% similarity levels was performed using the RDP classifier Bayesian algorithm (version 2.2) (Wang et al., 2007). Data associated with the soil bacteria and fungi were compared with the Silva bacterial database (http://unite.ut.ee/index.php) and the Unite reference database (http://unite.ut.ee/index.php). Subsequent analyses were based on the homogenized (minimum sample size) and flat data.

### Statistical Analysis and Graphing

The Wilcoxon rank-sum test was used to analyze whether the α-diversity indexes determined by Mothur (version v.1.30.2) and microbial community relative abundances were significantly different between groups (*p* <0.05). R (version 3.3.1) software was used to achieve the Venn diagram at the OTU level. Based on the results of the taxonomic analysis, the species compositions at the phylum and order levels of different groups were analyzed. Bray–Curtis distance matrices based on OTUs were initially calculated by Qiime 2, and then R (version 3.3.1) software (vegan package) was used to perform non-metric multidimensional scaling analysis (NMDS). In addition, linear discriminant analysis effect size (LEfSe) was used to identify microbial biomarkers (LDA > 3.5, *P* <0.05). Redundancy analysis (RDA) and canonical correspondence analysis (CCA) were performed in R (3.3.1) software (vegan package) based on OTU level after detrended correspondence analysis (DCA) to analyze the relationship between soil chemical properties and soil microorganisms. If the length of the gradient on the first axis of DCA results was ≥3.5, CCA was performed; if the value was <3.5, RDA was performed. Spearman's correlation coefficients were calculated between soil chemical properties and species at the order level, and the resulting matrix was visualized as a heatmap plot using R software (version 3.3.1) (heatmap package). FAPROTAX (version 1.1) was used to predict bacterial functional capabilities, and FUNGuild (version 1.0) was applied to predict the functions of fungi. The analysis methods mentioned above can be obtained from the free online Majorbio Cloud Platform (https://cloud.majorbio.com/).

In addition, microbial co-occurring networks were constructed by calculating Spearman's correlation coefficients between OTUs with igraph (version 0.9.2). Soil chemical properties were analyzed by one-way ANOVA, and the Tukey–Kramer test was used to assess the significance of the groups (*P* <0.05). These tests were performed using SPSS software (version 20.0).

## Results

### α-Diversity and β-Diversity of Soil Bacteria and Fungi

**Figure 1:**
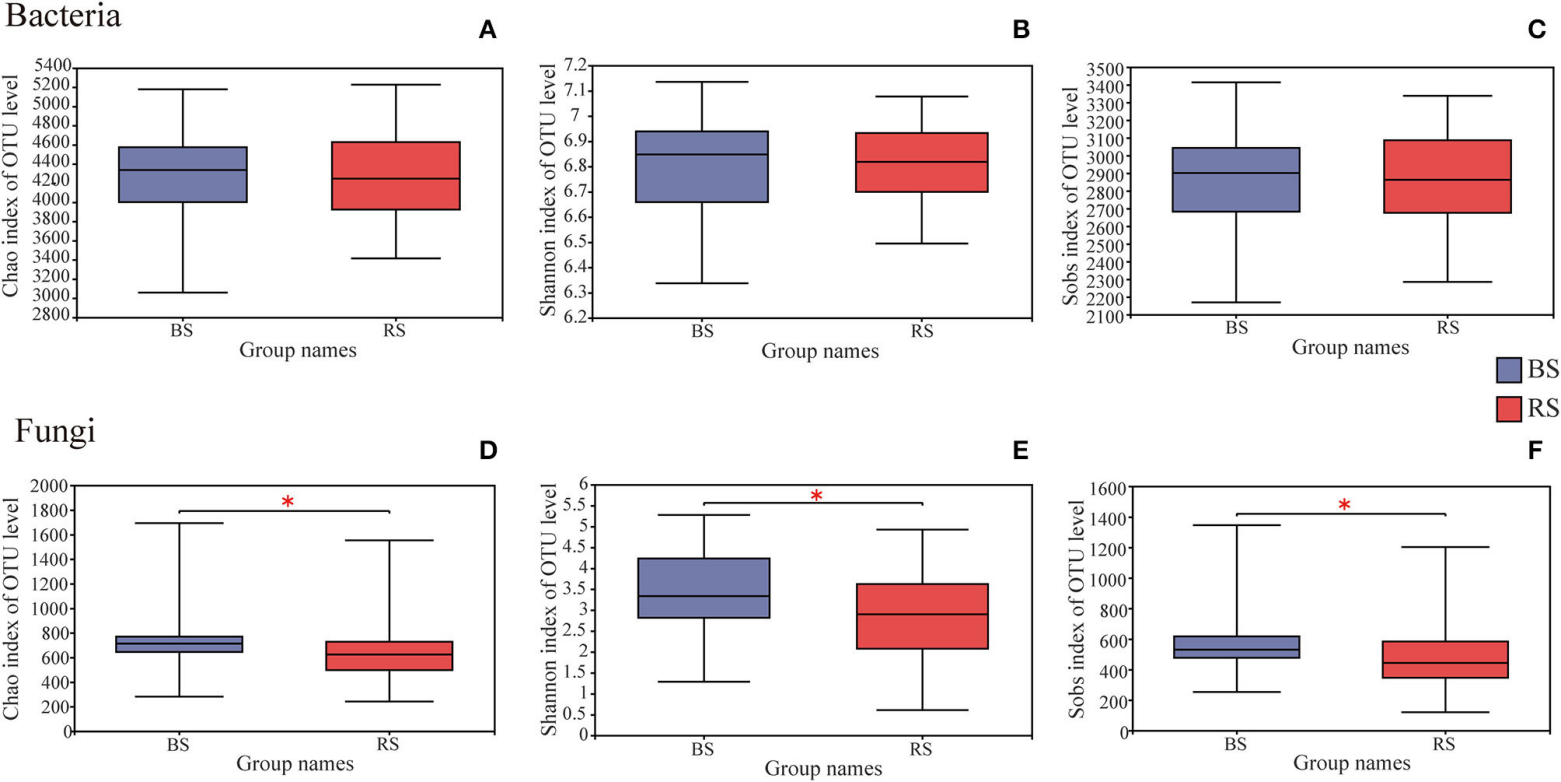
Differences in bacterial **(A–C)** and fungal **(D–F)** α-diversity indexes in the rhizosphere and bulk soils. The height and position of boxes were determined by upper and lower quartiles. The horizontal line inside boxes shows the median, while the outside top and bottom ones show the maximum and minimum values, respectively. *Indicates significant differences between two groups, *P* <0.05. BS and RS represent the bulk soil and the rhizosphere soil, respectively.

**Figure 2:**
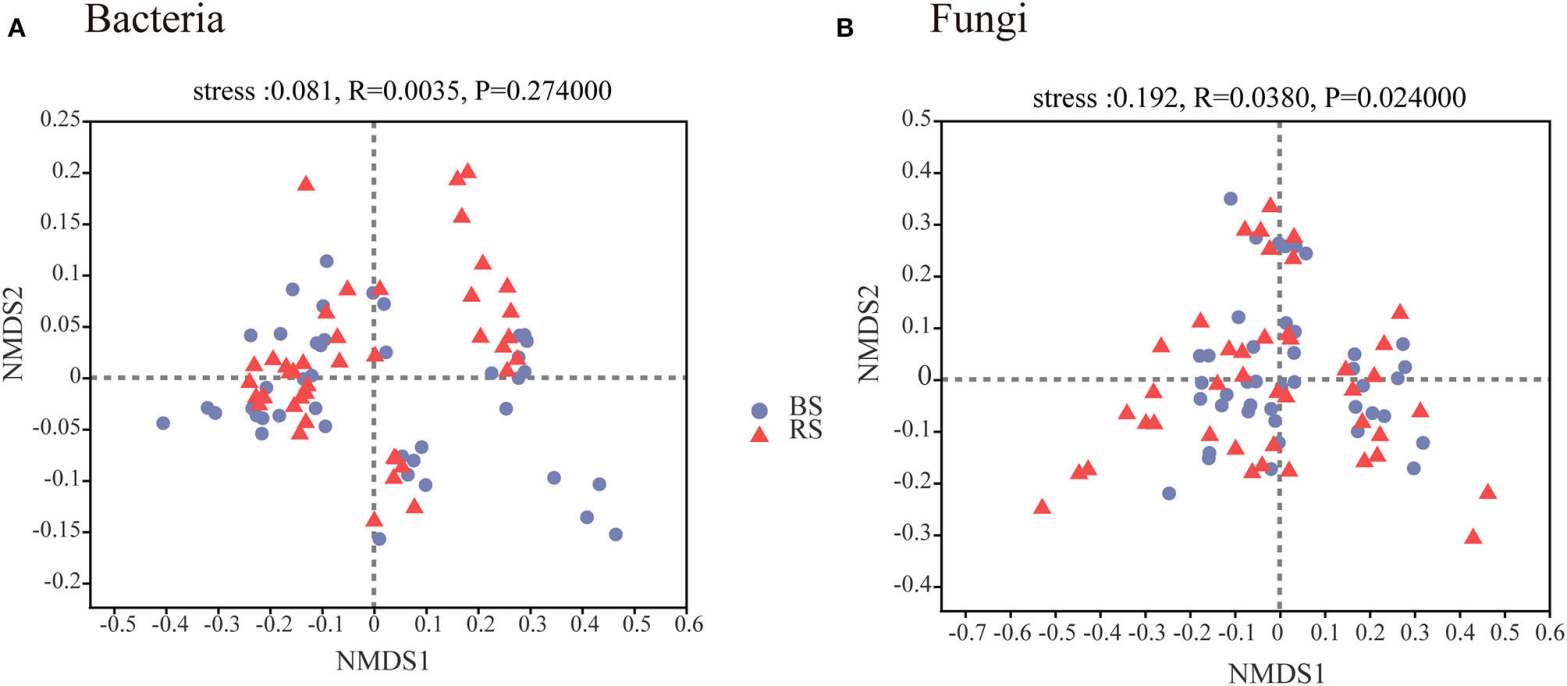
Bacterial **(A)** and fungal **(B)** β-diversity characteristics in the rhizosphere and bulk soils. NMDS of Bray–Curtis distances at OTU level. BS and RS, bulk soil and rhizosphere soil, respectively.

A total of 4,006,740 and 4,969,628 effective tags, and 1,669,331,778 and 1,259,286,659 total bases were identified from soil bacteria and fungi, respectively (Supplementary Table S1). The bulk soil and rhizosphere soil of pecan plantation had no obvious differences with regard to the α-diversity and β-diversity values of bacteria (Figures 1A–C, 2A). However, the fungal α-diversity and β-diversity values showed significant differences between the bulk soil and rhizosphere soil, and a higher α-diversity value was observed in the bulk soil (Figures 1D–F, 2B).

### Community Compositions of Soil Bacteria and Fungi

**Figure 3:**
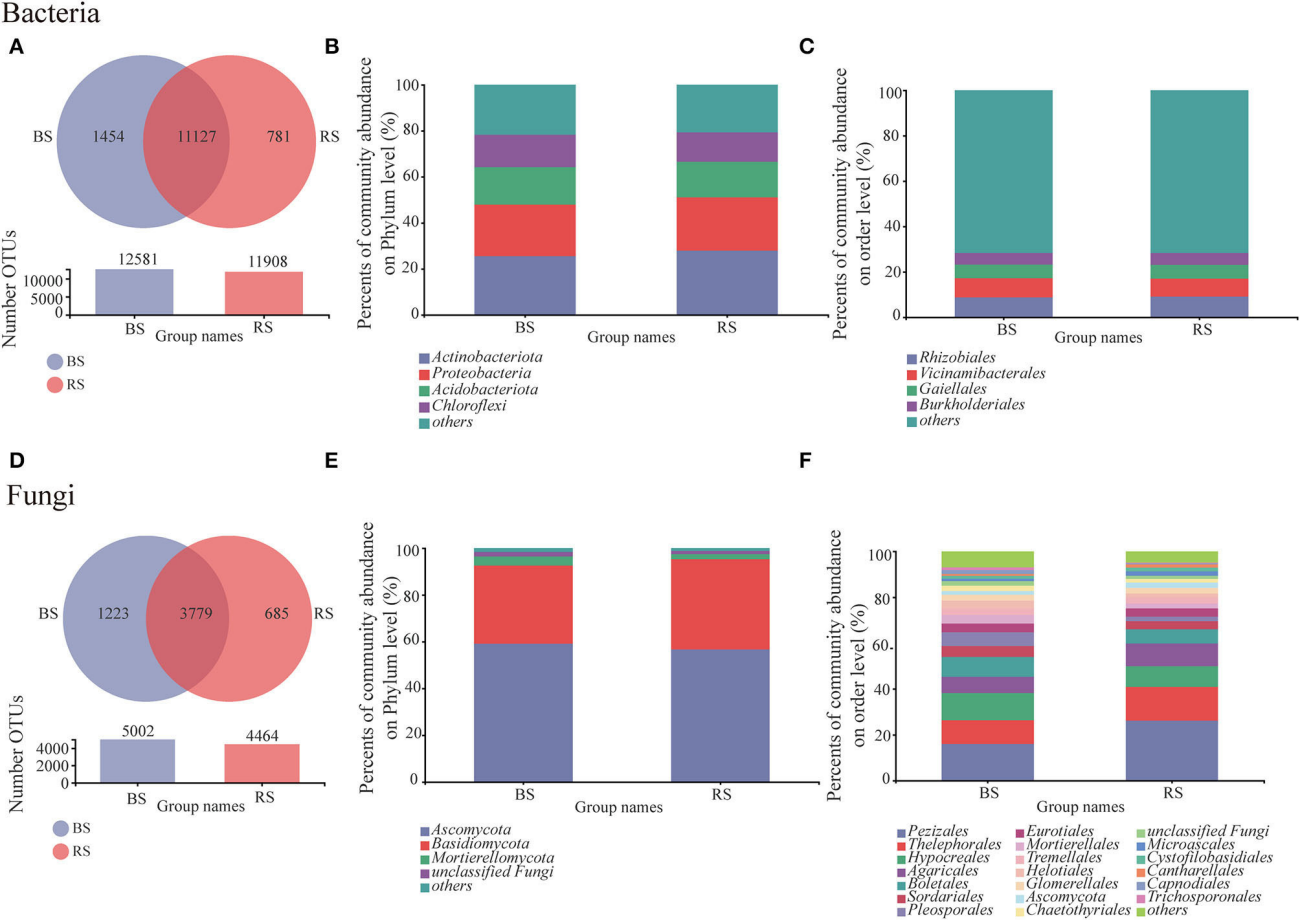
Bacterial and fungal community compositions in the rhizosphere and bulk soils. **(A,D)** Venn diagram for the OTU numbers. **(B,E)** and **(C,F)** relative abundances of bacteria/fungi at phylum and order levels, respectively. BS and RS, bulk soil and rhizosphere soil, respectively.

Bulk soil showed a higher OTU number than that observed in the rhizosphere soil, regardless of bacteria or fungi (Figures 3A,D). The shared bacterial and fungal OTU number between the bulk soil and rhizosphere soil accounted for 83.27 and 66.45% of the total OTU of bacteria and fungi, respectively (Figures 3A,D). Furthermore, OTU specific to rhizosphere bacteria and fungi accounted for 5.84 and 12.05%, respectively (Figures 3A,D). These results implied that although bacteria were superior in total OTU number, the host specificity of rhizosphere fungi was stronger than that of bacteria.

Obviously, the composition of bacterial communities in rhizosphere and bulk rhizosphere soils was similar, mainly composed of *Actinobacteria, Proteobacteria, Acidobacteria*, and *Chloroflexi* (Figure 3B). While the composition of fungal communities at the order level showed differences between the rhizosphere and bulk soils, which was mainly reflected in the higher relative abundances of *Pezizales, Thelephorales*, and *Pleosporales* in the rhizosphere soil (Figure 3F).

### Differences in Taxa and Biomarkers of Bacteria and Fungi in the Rhizosphere and the Bulk Soils

**Figure 4:**
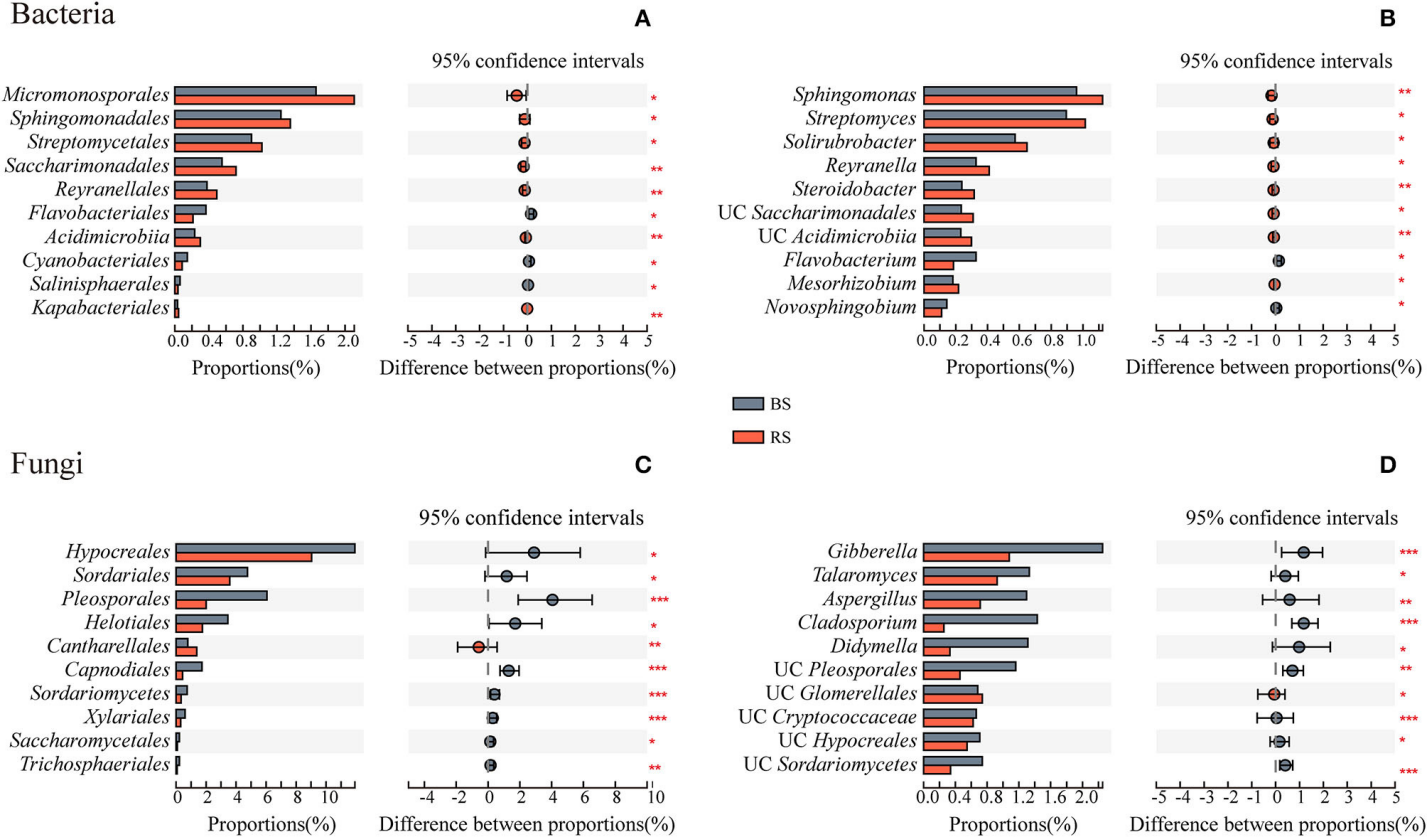
Taxa differences of bacteria and fungi between the bulk soil and the rhizosphere soil. **(A,C)** and **(B,D)** Show the microbial differences at the order and genus levels, respectively. The vertical axis shows taxa at order level with mean sums in the top 10, and different colored boxes indicate different groups. The horizontal axis represents the average relative abundance of taxa. *, **, and *** Indicate significant differences between the two groups, 0.01 < *p* <0.05, 0.001 < *p* <0.01, and *p* <0.001, respectively. BS and RS, bulk soil and rhizosphere soil, respectively. UC, unclassified.

Rhizosphere soil recruited more abundant bacteria of *Micromonosporales, Sphingomonadales, Streptomycetales, Saccharimonadales, Reyranellales, Acidimicrobiia*, and *Kapabacteriales* than the bulk soil (Figure 4A). They were mainly constituted by the genera *Sphingomonas, Streptomyces, Solirubrobacter, Reyranella*, and *Steroidobacter* (Figure 4B). However, except that the relative abundance of *Cantharellales* in the rhizosphere soil was significantly higher than that in the bulk soil, the relative abundances of most fungal taxa were significantly reduced in the rhizosphere soil environment, which is mainly reflected in the genera *Gibberella, Talaromyces, Aspergillus, Cladosporium*, and *Didymella* (Figures 4C,D).

**Figure 5:**
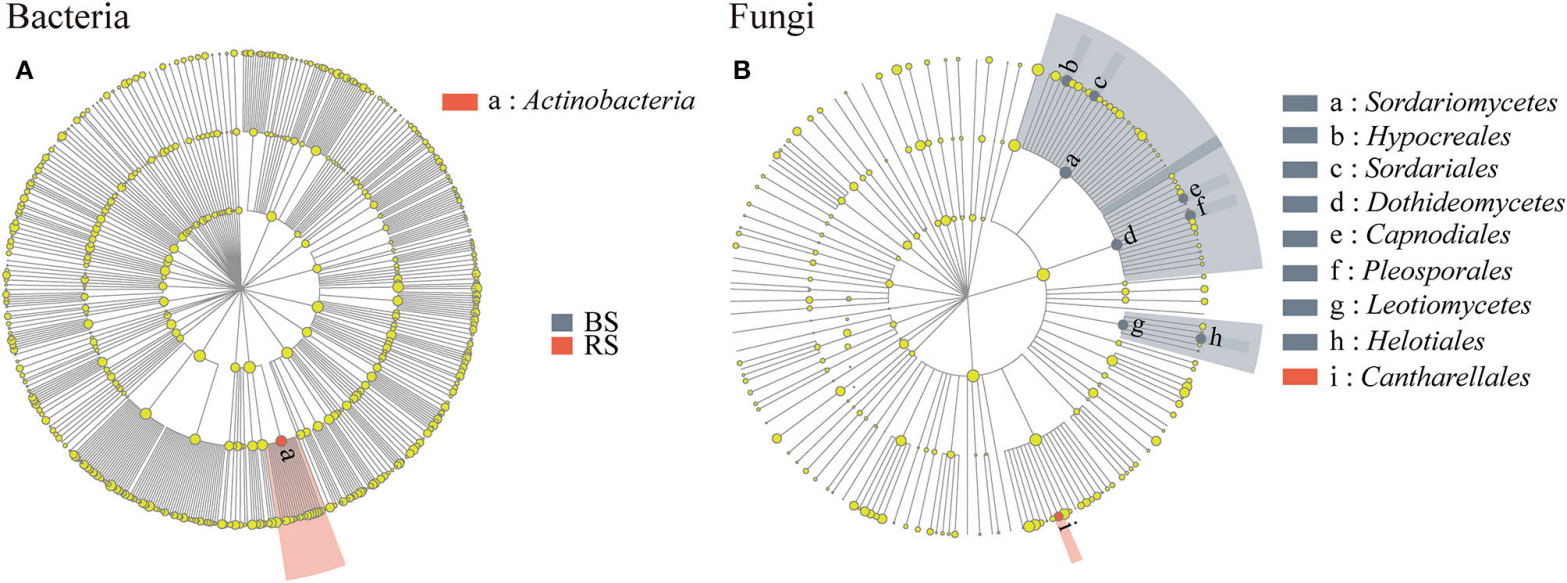
**(A,B)** LEfSe results in the bulk soil and the rhizosphere soil. Linear discriminant analysis (LDA) was carried out on samples according to different grouping conditions based on taxonomic compositions to identify communities or species that had a significant differential impact on sample delineation. The classification level of tree diagrams is from phylum to order. Node size, the relative abundances of each species. Different colored nodes, microorganisms that are significantly enriched in the corresponding group. Yellowish nodes, no significant differences between groups. LDA > 3.5 and *p* <0.05. BS and RS, bulk soil and rhizosphere soil, respectively.

Linear discriminant analysis effect size results showed that *Actinobacteria* and *Cantharellales* were the bacterial and fungal biomarkers of the rhizosphere soil of pecan plantation, respectively (Figures 5A,B). In addition, *Dothideomycetes, Hypocreales, Helotiales, Pleosporales, Sordariomycetes*, and *Leotiomycetes* were identified as the most important fungal biomarkers for the bulk soil (LDA ≥ 4.0) (Figure 5B; Supplementary Table S2).

### Relationship Between Soil Chemical Properties and Microorganisms

**Figure 6:**
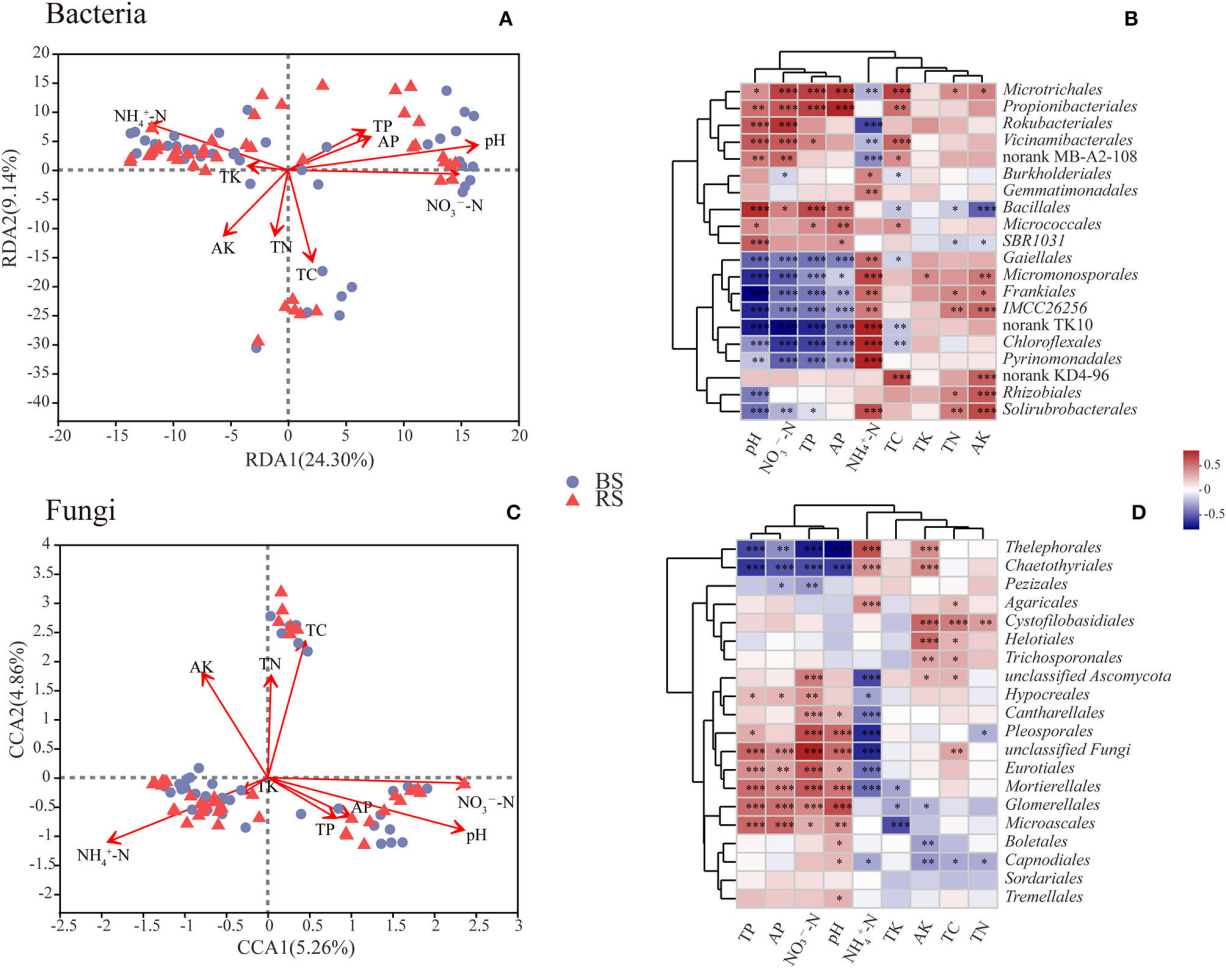
Correlations between microbial communities and soil chemical properties. **(A,C)** RDA/CCA results of the relationship between bacteria/fungi community structure and soil chemical properties. Different colored or shaped dots in the graph indicate groups of samples in different environments, and arrows indicate environmental factors. The length of the line between the arrow and the origin represents the degree of correlation between an environmental factor and the distribution of communities, and the longer the line, the higher the correlation. The angle between the arrow and the ranking axis represents the correlation between an environmental factor and the ranking axes, and the smaller the angle, the higher the correlation. BS and RS, bulk soil and rhizosphere soil, respectively. **(B,D)** Correlation analysis results between the relative abundance of dominant soil microorganisms at the order level and soil chemical properties. The graphs show the top 20 orders in the relative abundances. The horizontal and vertical axes show the soil chemical properties and species, respectively. Spearman's correlation coefficients are shown in different colors in the graphs. *, **, and *** Indicate 0.01 < *p* <0.05, 0.001 < *p* <0.01, and *p* <0.001, respectively. TC, TN, TP, and TK, total contents of carbon, nitrogen, phosphorus, and potassium, respectively; ${\text{NH}}_{4}^{+}$-N and ${\text{NO}}_{3}^{-}$-N, ammonium nitrogen and nitrate nitrogen; AP and AK, active contents of phosphorus and potassium, respectively.

The contents of total carbon (TC), total nitrogen (TN), total phosphorus (TP), active phosphorus (AP), and ammonium nitrogen (${\text{NH}}_{4}^{+}$-N) in the rhizosphere soil were significantly higher than those observed in the bulk soil (*p* <0.05) (Supplementary Table S3). However, the pH value in the rhizosphere soil showed a decrease compared to that noticed in the bulk soil (Supplementary Table S3). RDA or CCA results showed that the bacterial and fungal community structure was most strongly correlated with pH and the contents of ${\text{NH}}_{4}^{+}$-N and nitrate nitrogen (${\text{NO}}_{3}^{-}$-N) (Figures 6A,C). Additionally, the fungal community structure was also closely related to the TC content (Figure 6C).

Correlation heatmap showed that pH was significantly positively correlated with the relative abundances of *Microtrichales, Propionibacteriales, Rokubacteriales, Vicinamibacterales, Bacillales*, and *Micrococcales*, but was negatively related to the relative abundances of *Gaiellales, Micromonosporales, Frankiales, Chloroflexales, Pyrinomonadales*, and *Rhizobiales* (Figure 6B). These results indicated that the above-mentioned bacterial taxa have a preference for the pH of the habitat. Furthermore, the relative abundances of *Gaiellales, Micromonosporales, Frankiales, Chloroflexales*, and *Pyrinomonadales* had a negative relationship with the contents of ${\text{NO}}_{3}^{-}$-N, AP, and TP, but had a positive relationship with the content of ${\text{NH}}_{4}^{+}$-N (Figure 6B). Interestingly, *Microtrichales, Rokubacteriales*, and *Vicinamibacterales* showed an opposite correlation pattern with the above-mentioned taxa (Figure 6B). In addition, TC content was remarkably positively correlated with the relative abundances of *Microtrichales, Propionibacteriales, Vicinamibacterales*, and *Microtrichales*, but was notably negatively related with the relative abundances of *Burkholderiales, Bacillales, Gaiellales*, and *Chloroflexales* (Figure 6B). TN and active potassium (AK) contents were positively correlated with the abundances of *Microtrichales, Frankiales, Rhizobiales*, and *Solirubrobacterales*, however, were negatively related to the abundance of *Bacillales* (Figure 6B).

In terms of fungal community, the abundances of *Thelephorales, Chaetothyriales*, and *Pezizales* showed a negative correlation with the contents of TP, AP, ${\text{NO}}_{3}^{-}$-N, and pH, while they showed a positive relationship with the content of ${\text{NH}}_{4}^{+}$-N (Figure 6D). However, fungal taxa of *Hypocreales, Cantharellales, Pleosporales, Eurotiales, Mortierellales, Glomerellales*, and *Microascales* exhibited an opposite pattern (Figure 6D). Additionally, the contents of AK, TC, and TN had a positive relationship with the abundances of *Cystofilobasidiales, Helotiales*, and *Trichosporonales*, but had a negative correlation with the abundances of *Capnodiales* and *Glomerellales* (Figure 6D).

### Soil Microorganism Co-occurring Network Structures

**Figure 7:**
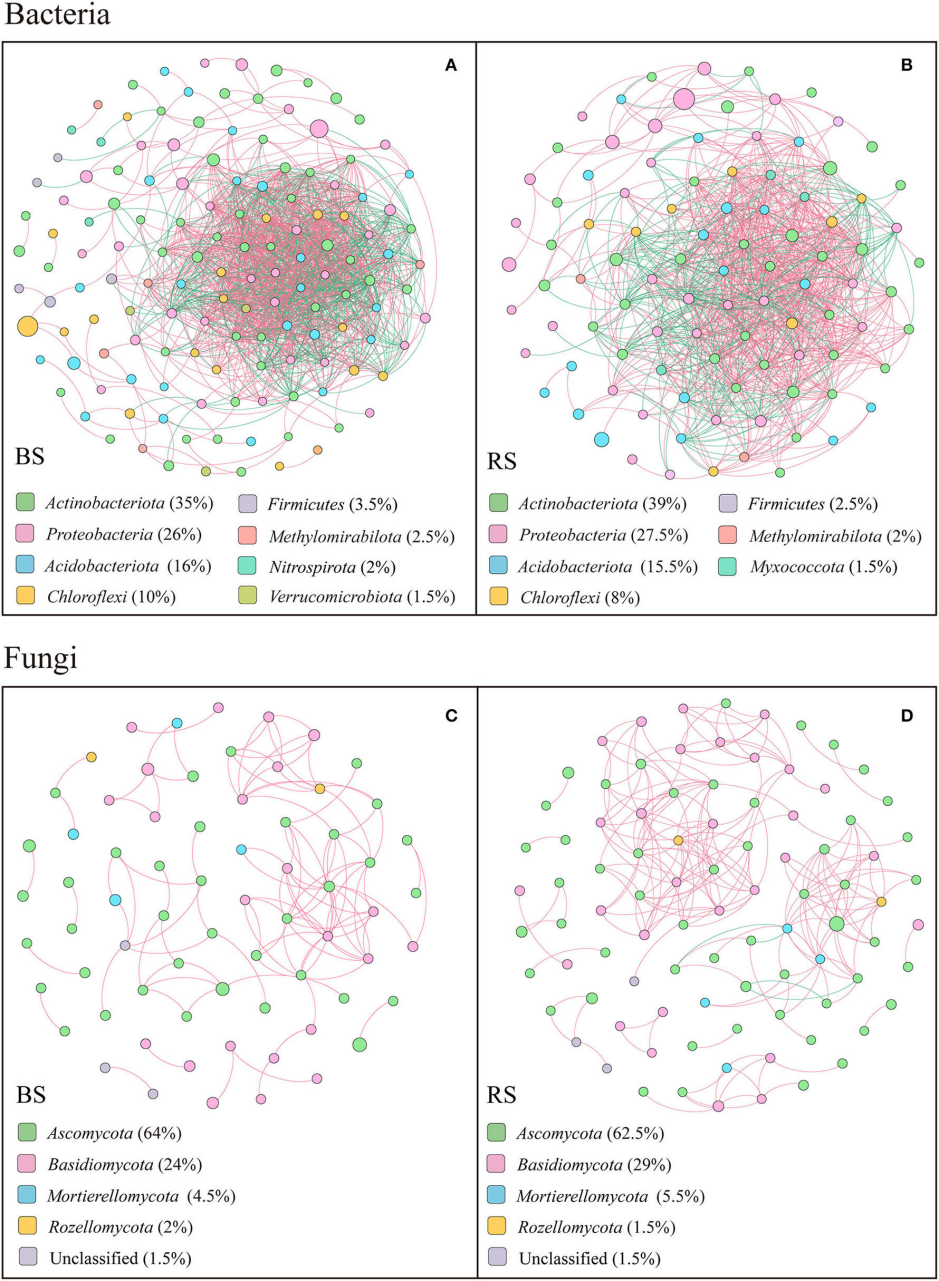
Results of correlation network analysis of bacteria **(A,B)** and fungi **(C,D)** in the bulk soil and the rhizosphere soil. The top 200 OTUs were selected, and Spearman's correlation coefficient rank between the relative abundances of OTUs was calculated to reflect the correlations. Only OTUs with strong significant correlations are shown in the figure (*p* <0.05 and Spearman's correlation coefficients size > 0.8). The size of nodes indicates the size of OTU abundance, and different colors indicate different phyla. Red connecting lines indicate a positive correlation, and blue lines indicate a negative correlation. The thicker the line, the higher the correlation between OTUs, and the more the number of lines, the closer the association between the taxa.

The co-occurring networks showed that the bacterial networks in the bulk and rhizosphere soils of pecan plantation had higher connectivity and node degree than the fungal networks (Figures 7A–D; Supplementary Tables S4–S6). In terms of the bacterial networks, bulk-soil network had more total nodes and links than the rhizosphere-soil network (Figures 7A,B; Supplementary Table S4). In addition, the number of positive links and negative links of bacteria each accounted for half of the total links, both in the bulk-soil and rhizosphere-soil networks (Supplementary Table S4). However, in the fungal networks, the rhizosphere-soil network showed more total nodes and links than the bulk-soil network, and total positive links were much greater than total negative links. Precisely, 108 positive links and 0 negative links of the fungal community were constructed in the bulk-soil network, while there were 211 positive links and 4 negative links in the rhizosphere-soil network (Supplementary Table S4). Additionally, compared to the bulk soil, the fungal rhizosphere-soil network had a higher average clustering coefficient (Supplementary Table S4).

The top 20 node degrees showed that the bacterial keystone taxa in the bulk soil were composed of four members of *Actinobacteriota*, three members of *Chloroflexi*, seven members of *Proteobacteria*, four members of *Acidobacteriota*, one member of *Methylomirabilota*, and one member of *Verrucomicrobiota*, while in the rhizosphere soil were composed of four members of *Proteobacteria*, two members of *Myxococcota*, six members of *Acidobacteriota*, seven members of *Actinobacteriota*, and one member of *Chloroflexi* (Supplementary Table S5). More specifically, *Acidothermus, Candidatus Solibacter, Gaiellales, Jatrophihabitans, Sphingomonas, Candidatus Udaeobacte, Vicinamibacteria, Rokubacteriales, Nitrosomonadaceae*, and *Burkholderiales* played an important role in the bulk-soil bacterial network, while *Elsterales, Acidothermus, Candidatus solibacter, Gaiellales, Nitrosomonadaceae, Micromonosporaceae, Phaselicystis, Ensifer*, and *Haliangium* were the keystone taxa in the rhizosphere-soil network (Supplementary Table S5).

In both bulk soil and rhizosphere soil, the keystone taxa in the fungal network consisted mainly of *Ascomycetes* (Supplementary Table S6), specifically *Fusicolla, Fusarium, Arthropsis, Tuber, Thielavia, Fusicolla, Arthrographis*, and *Pyrenochaetopsis* as the bulk-soil keystone genera, and *Pyrenochaetopsis, Sphaerosporella, Cladophialophora, Chaetomium, Dichotomopilus*, and *Tuber* as the rhizosphere-soil keystone genus (Supplementary Table S6). In addition, members of *Basidiomycota*, including *Cutaneotrichosporon, Cystofilobasidium*, and *Cryptococcus*, and members of *Mortierellomycota*, including *Mortierella*, were the keystone taxa in the bulk-soil network. The members of *Basidiomycota*, including *Cutaneotrichosporon, Tomentella, Clavulina, Cystofilobasidium*, and *Trechispora*, members of *Mortierellomycota*, including *Mortierella*, and unclassified *Rozellomycota* played vital functions in the rhizosphere-soil network (Supplementary Table S6).

### Prediction of Soil Bacterial and Fungal Functions

**Figure 8:**
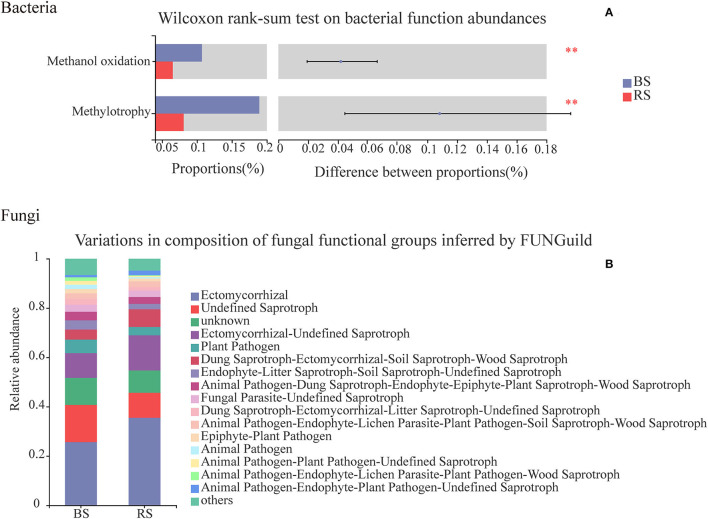
Functional predictions of bacteria and fungi in the bulk soil and the rhizosphere soil. **(A)** Vertical axis, function names; horizontal axis, the percentage values of the functional abundances. Different colors indicate different groups. **Indicate *p* <0.01. The top nine functional abundances are shown. **(B)** Vertical axis, the proportion of Guild's abundance in different groups; horizontal axis, different groups.

Chemoheterotrophy, animal parasites or symbionts, human pathogens, nitrate reduction, nitrogen fixation, cellulolysis, aromatic compound degradation, and predatory or exoparasitic process were the main functions of the soil bacteria of pecan plantation (Supplementary Table S7). Compared to the bulk-soil bacteria, the rhizosphere-soil bacteria had the stronger capabilities of chemoheterotrophy, nitrogen fixation, cellulolysis, and aromatic compound degradation (Supplementary Table S7). However, the rhizosphere-soil bacteria showed a significant decrease in the functions of methanol oxidation and methylotrophy, in contrast to the bulk-soil bacteria (Figure 8A), and the relative abundance of bacterial pathogens in the rhizosphere soil also decreased (Supplementary Table S7).

Functional relative abundances of soil fungi in the pecan plantation differed between the bulk soil and the rhizosphere soil (Figure 8B). The ectomycorrhizal function was the predominant of all the fungal functions, and accounted for 25 and 35% in the bulk soil and the rhizosphere soil, respectively (Figure 8B). Additionally, fungi in the rhizosphere soil had a higher functional relative abundance of wood saprotrophs and lower functional relative abundances of human and plant pathogens, in contrast to the functional relative abundances of fungi in the bulk soil (Figure 8B).

## Discussion

### Rhizosphere-Associated Fungal Rather Than Bacterial Community Structures Differed From Bulk Soil in Pecan Plantations

Previous studies documented dramatic differences between the bulk and rhizosphere soils with regard to the bacterial community in the terrestrial ecosystems (Mendes et al., 2014; Fan et al., 2017; Essel et al., 2019). However, we found that fungi rather than bacteria showed significant differences in the diversity and community composition between the bulk soil and the rhizosphere soil, and a higher fungal diversity was observed in the bulk soil (Figures 1–3). We speculated that fungi, especially mycorrhizal fungi, are generally more deeply associated with plants than bacteria (Buée et al., 2009). Rhizosphere soil of pecan plantations had a preference for recruiting specific fungal communities rather than bacteria due to the higher sensitivity of specific fungi to the molecular signals from pecan root secretions (Badri et al., 2009; Baetz and Martinoia, 2014; Spence and Bais, 2015). These speculations need to be confirmed by further research. Our results also verified that fungi showed stronger rhizosphere effects than bacteria for rhizosphere-specific OTU ratios (Figures 3A,D). This observation may be specific to the rhizosphere of pecan plants. We speculated that there was an obvious selection at the taxonomic level in the assembly process of the pecan rhizosphere community, which led to a lower α-diversity index of rhizosphere fungi compared to the bulk soil. This selection largely follows the niche-based theory (Mendes et al., 2014). It was reported that niche filtering would occur in the rhizosphere microbial community assembling, while the bulk-soil microbial community preferred to be regulated by neutral processes (Mendes et al., 2014).

Similar to the studies in *Camellia oleifera* (Zhang et al., 2020), *Platycladus orientalis* (Liu et al., 2018), and grasslands (Nan et al., 2020), A*ctinobacteria, Proteobacteria, Acidobacteria*, and *Chloroflexi* mostly dominated in our pecan plantations, and *Ascomycota* and *Basidiomycota* were the dominant fungal phyla. This finding is related to the widespread dispersal capabilities, lifestyles, and functional attributes of these species, and most members of these dominant bacteria and fungi have the genomic potential for higher competition, resource utilization, and stress tolerance, which contributes to their increased dominance in various soils (Egidi et al., 2019).

### Bacterial and Fungal Differential Taxa and Biomarkers in the Bulk and Rhizosphere Soils of the Pecan Plantations

To maintain the rhizosphere health and promote plant development, specific beneficial microorganisms could be recruited to improve nutrient acquisition and combat pathogenic taxa (Fan et al., 2017). In the bacterial community, we found *Sphingomonas, Streptomyces, Solirubrobacter, Reyranella*, and *Steroidobactery* were enriched in the rhizosphere soil of pecan plantations (Figure 4B), which could be attributed to the rhizosphere effect. These taxa were widely reported as the plant growth-promoting rhizobacteria (PGPR) involved in pathogen control, nitrogen fixation, and phytohormone production (Prashar et al., 2013; Mendes et al., 2014; Franke-Whittle et al., 2015).

In the fungal community, we found that higher relative abundances of *Pezizales, Thelephorales*, and *Pleosporales* occurred in the rhizosphere soil (Figure 3F). The population of *Pezizales* was found to be enriched in the rhizosphere soil, since the pecan tree was its natural host. *Truffle*, fruiting bodies of *Pezizales* with high economic value, was the focus of our research team in the later stage. Interestingly, we also found *Cantharellales* was the fungal biomarker of the rhizosphere soil (Figure 5B). *Chanterelle* is an edible fungus with economic, medicinal, and ecological functions, which can symbiotically grow with plants to form ectomycorrhizae (Karwa et al., 2011). It plays an important role in the natural material cycle (Chang and Miles, 2004) and effectively controls the soil-borne diseases of the host plant (Chang and Miles, 2004). The multifunctional role of *Chanterelle*, as the fungal biomarker for the rhizosphere of pecan plantation, recruitment, function, and evolution of *Chanterelle*-associated microorganisms, needs to be further explored. Members of *Thelephorales* were usually recognized as the ectomycorrhizal fungi, and evidence had been provided for the ectomycorrhizal formation between tropical forest plants and *Thelephorales* (Yorou, 2008; Looby and Eaton, 2014; Bauman et al., 2016). *Pleosporales* occur in various habitats as endophytes, epiphytes, or saprobes of dead plants, and are mainly involved in saprophylaxis and plant pathogenic processes (Zhang Y. et al., 2012). According to the microbial interaction theory, the intervention of pathogenic fungi will lead to the existence of more probiotics than antagonize it (Frey-Klett et al., 2011), which can explain the enrichment of anti-pathogenic beneficial bacteria in the rhizosphere of pecan plantation.

The interaction between soil physicochemical characteristics and rhizodeposits drives the differential niches and exerts niche forces in the microbial community assembly (Mendes et al., 2014). Our results verified the previous reports that the rhizosphere of cultivated plants prefers selecting beneficial microorganisms (Dibbern et al., 2014; Mendes et al., 2014). In addition, pecan trees are natural hosts for a variety of edible fungi, which opens up a new avenue for the exploitation of beneficial fungal resources.

### Important Soil Factors Affecting Microbial Community Structure

Previous studies documented that pH had a strong relationship with bacterial and fungal community structures (Essel et al., 2019; Liu C. et al., 2021; Shu et al., 2021), and the results of this study confirmed that pH was also a very important driving factor of microbial communities in bulk and rhizosphere soils of pecan trees. Additionally, ammonium nitrogen (${\text{NH}}_{4}^{+}$-N) and nitrate nitrogen (${\text{NO}}_{3}^{-}$-N) contents were also closely related to the structure of the bacterial community (Figure 6A), while total carbon (TC) content had a strong relationship with the structure of the fungal community (Figure 6C). These results could be attributed to the fact that soil bacteria of pecan plantations, such as *Gaiellales* and *Frankiales* which significantly positively correlated with ${\text{NH}}_{4}^{+}$-N content, are mainly involved in N cycling, while soil fungi, such as *Cystofilobasidiales, Helotiales*, and *Trichosporonales* which had a remarkable positive relationship with TC, mainly related to C cycling. It was noteworthy that the mycorrhizal fungi like *Thelephorales* and *Pezizales*, which enriched in the rhizosphere soil, exhibited a significant positive correlation with ${\text{NH}}_{4}^{+}$-N content, and the ${\text{NH}}_{4}^{+}$-N content in the rhizosphere soil was significantly higher than that observed in the bulk soil. These results indicated that the members of mycorrhizal fungi inhabiting the rhizosphere of pecan plantation may be more willing to participate in the nitrogen fixation.

### Microbial Network Structures and Ecological Functions

As expected, bacterial and fungal network properties differed between the bulk and rhizosphere soils (Figure 7; Supplementary Tables S4–S6). The bacterial network in the bulk soil of the pecan plantation had more total nodes and links than in the rhizosphere soil (Supplementary Table S4), which indicated rhizosphere bacteria had a simpler network than the bacteria of the bulk soil. These results could be attributed to the lower diversity of rhizosphere soil which showed a less dynamic structure and stronger ecological stability (Fan et al., 2018). However, the fungal rhizosphere effect on the ecological network showed the opposite pattern, and a more complex network of rhizosphere network was exhibited (Figure 7; Supplementary Table S4), which was related to the greater influence of rhizosphere effects on fungal diversity and composition, to facilitate a more close association of the rhizosphere with fungi than with bacteria (Wang G. et al., 2021). Interestingly, a 50/50 split between positive and negative correlations was shown in the bacterial networks, while links in the fungal network mostly showed a positive relationship (Supplementary Table S4). We speculated bacterial interactions were more complex, and thus had strong network patterns of co-occurrence and mutual exclusion. However, fungi were more open to establishing win-win partnerships with each other (Miransari, 2011), such that fungi dominated the co-occurrence network pattern. Microbial ability to use organic carbon would affect the structure of microbial communities (Zhu et al., 2021). Bacteria usually dissolved organic carbon more efficiently, while fungi preferred complex organic carbon compounds, such as lignin and cellulose (Zhu et al., 2021). Compared to bacteria, fungi usually respond more rapidly to carbon rhizodeposits (Zhu et al., 2021). Our results showed that the relationship between fungi and TC was stronger than that between bacteria and TC content, while *Helotiales* and *Capnodiales*, which were the fungal biomarkers for bulk soil, had strong positive correlations with the TC content (Figures 5B, 6D). Saprophytic fungi play a vital role in litter decomposition in forests and are closely linked to the quality of apoplastic litter (Liu C. et al., 2021). We considered that *Helotiales* and *Capnodiales* altered the fungal community structure, and led to differences between the bulk-soil and rhizosphere-soil fungal networks due to the higher litter quantity in the bulk soil which increased TC content.

In the bacterial and fungal networks, we found a number of key species (Supplementary Tables S5, S6). Bacterial keystone species, including *Acidothermus, Solibacter, Gaiellales, Jatrophihabitans, Sphingomonas, Candidatus Udaeobacte, Vicinamibacteria, Rokubacteriales, Nitrosomonadaceae, Burkholderiales, Elsterales, Micromonosporaceae, Phaselicystis, Ensifer*, and *Haliangium*, are mostly recognized as PGPRs and involved in carbon and nutrient cycling (Mendes et al., 2014; Lopez-Lozano et al., 2020; Ayangbenro and Babalola, 2021; He et al., 2021; Le Roux et al., 2021; Signorini et al., 2021). Among the different fungal groups, mycorrhizal fungi are considered to be important symbionts of plants, and saprophytic fungi contribute to carbon mineralization in the soil and are the main decomposers in forest litter (Liu C. et al., 2021). We found *Thielavia*, with the ability to decompose lignin and cellulose, was one of the fungal keystone species. *Tomentella* and *Tuber* were also identified as keystone taxa in the fungal networks (Supplementary Table S6). Previous studies verified that pecan trees can form ectomycorrhizal associations with a wide range of fungi, including *Tomentella* and *Tuber*, whose substrates are edible and have nutritional or medicinal value (Freiberg et al., 2021; Grupe et al., 2021; Habtemariam et al., 2021). Despite the dominance of many beneficial species in bacterial and fungal networks had been found, some pathogenic fungi still need to be noticed in the management of pecan plantations, such as the bulk-soil keystone genus of *Fusarium* and the rhizosphere-soil genus of *Cladophialophora* in this study, which were usually reported as the plant pathogens and environmental saprobes (Badali et al., 2008; Rampersad, 2020; He et al., 2021).

Our results for predicting the function of bacteria and fungi showed that compared to the bulk-soil bacteria, the rhizosphere-soil bacteria had the stronger capabilities of chemoheterotrophy, nitrogen fixation, cellulolysis, and aromatic compound degradation (Supplementary Table S7). We speculated that the recruitment of specific microorganisms by root secretions distinguished the rhizosphere bacterial community from that of the bulk soil, particularly as the increase in aromatic substrates secreted by the root system of pecans enhanced the functional relative abundances of the rhizosphere bacteria in the decomposition of aromatic compounds. Due to the enrichment of plant-promoting bacteria, such as nitrogen-fixing bacteria in the rhizosphere network, the functional abundance of nitrogen fixation and other bacteria involved in chemical element cycling was higher in the rhizosphere soil than in the bulk soil. In addition, similar results were reported by Wang G. et al. (2021), and we found the microbial structure of rhizosphere soil was healthier than bulk soil (Figure 8; Supplementary Table S7).

## Conclusion

Rhizosphere-associated fungal rather than bacterial community structures differed from bulk soil in pecan plantations. The pH value and the content of ammonium nitrogen (${\text{NH}}_{4}^{+}$-N), nitrate nitrogen (${\text{NO}}_{3}^{-}$-N), and total carbon (TC) were the most important drivers of microbial community structure and composition. Simpler bacterial network structures occurred in the pecan rhizosphere than in the bulk soil, which was opposite to fungal network patterns. Plant growth-promoting rhizobacteria (PGPR), which were enriched in the rhizosphere soil, are mainly involved in protection against pathogens or nitrogen fixation. In addition, the pecan rhizosphere could become the repository for tapping edible mycorrhizal fungi. This study provides insights into the drivers of microbial community assembly in rhizosphere and bulk soils and lays a theoretical and practical basis for the sustainable management of pecan plantations.

## Data Availability Statement

The datasets presented in this study can be found in online repositories. The names of the repository/repositories and accession number(s) can be found in the article/Supplementary Material.

## Author Contributions

JL: conceptualization, methodology, data curation, visualization, writing of the original draft, reviewing, and editing. YT, JB, and HW: data curation, reviewing, and editing. FP: conceptualization and funding acquisition. PT: methodology and supervision. GC and SL: data curation. All authors contributed to the article and approved the submitted version.

## Funding

This work was funded by the National Key Research and Development Project of China (2018YFD1000604), the Postgraduate Research and Practice Innovation Program of Jiangsu Province (KYCX21_0911), and the Priority Academic Program Development of Jiangsu Higher Education Institutions (PAPD).

## Conflict of Interest

The authors declare that the research was conducted in the absence of any commercial or financial relationships that could be construed as a potential conflict of interest.

## Publisher's Note

All claims expressed in this article are solely those of the authors and do not necessarily represent those of their affiliated organizations, or those of the publisher, the editors and the reviewers. Any product that may be evaluated in this article, or claim that may be made by its manufacturer, is not guaranteed or endorsed by the publisher.
