# Supplementary material for: A Stronger Rhizosphere Impact on the Fungal Communities Compared to the Bacterial Communities in Pecan Plantations
Source: Front Microbiol. 2022 Jun 30;13:899801. doi: 10.3389/fmicb.2022.899801 (PMC9279573; doi:10.3389/fmicb.2022.899801)
Supplement: Supplementary file 1 [file Data_Sheet_1.doc]

**
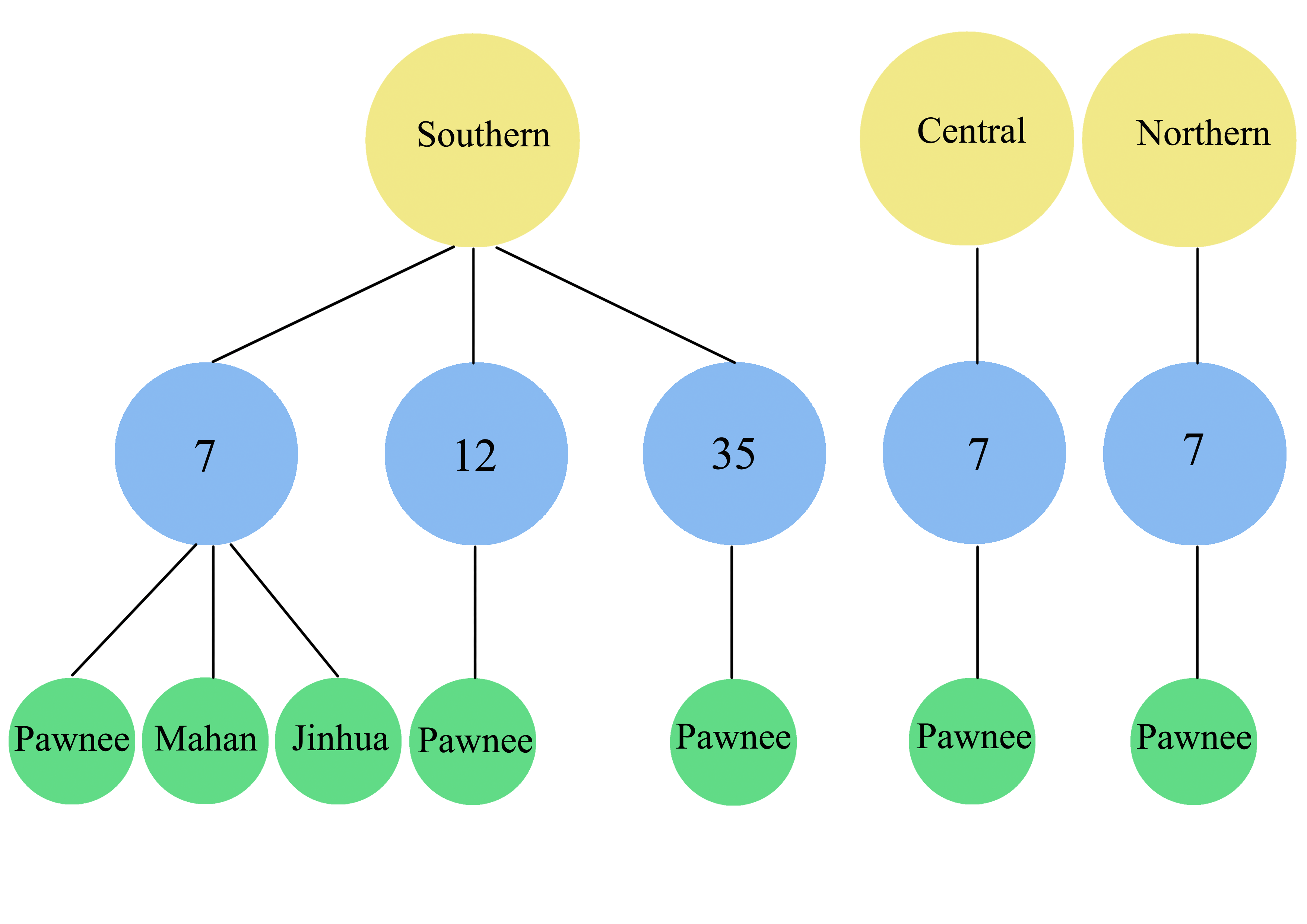
**

**Figure S1** Schematic diagram of nested experiment design

Yellow, blue and green circles indicate sampling areas (northern, central and southern Jiangsu Province), ages of pecan plantations (7-year-old, 12-year-old and 35-year-old) and pecan varieties (‘Pawnee’, ‘Mahan’ and ‘Jinhua’), respectively. 6 replicates were set in each subgroup, and a total of 42 rhizosphere soils and 42 bulk soils were collected.

**Table S1** Statistics for each sample of sequencing information

|  |  | Bacteria | | | Fungi | | |
| --- | --- | --- | --- | --- | --- | --- | --- |
| Sample ID | Groups | Effective tags | Total bases | Average length | Effective tags | Total bases | Average length |
| 1 | BS | 52880 | 21982169 | 416 | 71895 | 17342778 | 241 |
| 2 |  | 42173 | 17554008 | 416 | 70176 | 18267040 | 260 |
| 3 |  | 47073 | 19570037 | 416 | 70135 | 18542063 | 264 |
| 4 |  | 46421 | 19319756 | 416 | 36814 | 9080829 | 247 |
| 5 |  | 49967 | 20752827 | 415 | 51421 | 13889601 | 270 |
| 6 |  | 45907 | 19132412 | 417 | 72687 | 17611550 | 242 |
| 7 |  | 68924 | 28658708 | 416 | 74858 | 18511979 | 247 |
| 8 |  | 42686 | 17795621 | 417 | 61637 | 15106035 | 245 |
| 9 |  | 50463 | 21016894 | 416 | 54604 | 14061499 | 256 |
| 10 |  | 56481 | 23528727 | 417 | 52949 | 13310413 | 251 |
| 11 |  | 43916 | 18251840 | 416 | 45924 | 12680482 | 276 |
| 12 |  | 44932 | 18735617 | 417 | 56058 | 14258024 | 254 |
| 13 |  | 42691 | 17799161 | 417 | 68824 | 16902275 | 246 |
| 14 |  | 50425 | 21075274 | 418 | 43658 | 12167091 | 279 |
| 15 |  | 52912 | 21988210 | 416 | 60761 | 16068433 | 264 |
| 16 |  | 47251 | 19629621 | 415 | 51351 | 13715333 | 267 |
| 17 |  | 34856 | 14566131 | 418 | 59448 | 14528308 | 244 |
| 18 |  | 49874 | 20784909 | 417 | 65717 | 15634135 | 238 |
| 19 |  | 46188 | 19271780 | 417 | 55455 | 14085903 | 254 |
| 20 |  | 47549 | 19828923 | 417 | 54846 | 14796064 | 270 |
| 21 |  | 50224 | 20959610 | 417 | 59948 | 15068417 | 251 |
| 22 |  | 45787 | 19092306 | 416 | 51202 | 12784020 | 250 |
| 23 |  | 35810 | 14934472 | 417 | 49089 | 12627998 | 257 |
| 24 |  | 42117 | 17591174 | 418 | 56377 | 13963344 | 248 |
| 25 |  | 58226 | 24292445 | 417 | 63982 | 15505102 | 242 |
| 26 |  | 49169 | 20521723 | 417 | 71938 | 17232078 | 2340 |
| 27 |  | 38083 | 15902980 | 418 | 64787 | 15280084 | 236 |
| 28 |  | 40703 | 16988421 | 417 | 56492 | 13822741 | 245 |
| 29 |  | 44568 | 18642724 | 418 | 58817 | 16366632 | 278 |
| 30 |  | 56101 | 23490621 | 419 | 43689 | 13090602 | 300 |
| 121 |  | 42691 | 17799161 | 417 | 68824 | 16902275 | 246 |
| 122 |  | 50425 | 21075274 | 418 | 43658 | 12167091 | 279 |
| 123 |  | 52912 | 21988210 | 416 | 60761 | 16068433 | 264 |
| 124 |  | 47251 | 19629621 | 415 | 51351 | 13715333 | 267 |
| 125 |  | 34856 | 14566131 | 418 | 59448 | 14528308 | 244 |
| 126 |  | 49874 | 20784909 | 417 | 65717 | 15634135 | 238 |
| 145 |  | 52275 | 21570017 | 413 | 66712 | 16252226 | 244 |
| 146 |  | 36116 | 14984019 | 415 | 69519 | 17443174 | 251 |
| 147 |  | 51952 | 21676220 | 417 | 71161 | 16599509 | 233 |
| 148 |  | 48625 | 20220287 | 419 | 72370 | 17756117 | 245 |
| 149 |  | 40960 | 17102248 | 418 | 72437 | 17256286 | 238 |
| 150 |  | 40382 | 16858991 | 417 | 70226 | 16500779 | 235 |
| 31 | RS | 68924 | 28658708 | 416 | 74858 | 18511979 | 247 |
| 32 |  | 42686 | 17795621 | 417 | 61637 | 15106035 | 245 |
| 33 |  | 50463 | 21016894 | 416 | 54604 | 14061499 | 256 |
| 34 |  | 56481 | 23528727 | 417 | 52949 | 13310413 | 251 |
| 35 |  | 43916 | 18251840 | 416 | 45924 | 12680482 | 276 |
| 36 |  | 44932 | 18735617 | 417 | 56058 | 14258024 | 254 |
| 37 |  | 56328 | 23360359 | 415 | 54858 | 14748073 | 2689 |
| 38 |  | 53802 | 22358300 | 416 | 73990 | 19024092 | 257 |
| 39 |  | 67392 | 28072458 | 417 | 58628 | 15791104 | 269 |
| 40 |  | 40710 | 16943144 | 416 | 54241 | 14226722 | 262 |
| 41 |  | 40445 | 16824080 | 416 | 58726 | 16584524 | 282 |
| 42 |  | 36252 | 15082829 | 416 | 56559 | 14811451 | 262 |
| 43 |  | 41076 | 17098771 | 416 | 56715 | 14713242 | 259 |
| 44 |  | 44921 | 18659966 | 415 | 65358 | 16137027 | 247 |
| 45 |  | 50848 | 21146254 | 416 | 55023 | 14245234 | 259 |
| 46 |  | 50704 | 21091441 | 416 | 40158 | 9990548 | 249 |
| 47 |  | 45729 | 19011312 | 416 | 47888 | 12401186 | 259 |
| 48 |  | 40725 | 16953106 | 416 | 60293 | 14830382 | 246 |
| 49 |  | 59066 | 24591075 | 416 | 68794 | 15841223 | 230 |
| 50 |  | 46821 | 19568004 | 418 | 56150 | 14130199 | 252 |
| 51 |  | 53263 | 22213963 | 417 | 45668 | 11773055 | 258 |
| 52 |  | 53101 | 22144794 | 417 | 52175 | 12700488 | 243 |
| 53 |  | 49353 | 20507843 | 416 | 49526 | 12940595 | 261 |
| 54 |  | 49419 | 20587963 | 417 | 74914 | 17747631 | 237 |
| 55 |  | 52849 | 22025399 | 417 | 50400 | 12383588 | 246 |
| 56 |  | 50887 | 21210088 | 417 | 57008 | 14355960 | 252 |
| 57 |  | 46057 | 19257425 | 418 | 72092 | 17823588 | 247 |
| 58 |  | 52331 | 22014159 | 421 | 46349 | 11817267 | 255 |
| 59 |  | 47596 | 19928326 | 419 | 59976 | 15948450 | 266 |
| 60 |  | 46423 | 19416231 | 418 | 60126 | 16123980 | 268 |
| 127 |  | 46188 | 19271780 | 417 | 55455 | 14085903 | 254 |
| 128 |  | 47549 | 19828923 | 417 | 54846 | 14796064 | 270 |
| 129 |  | 50224 | 20959610 | 417 | 59948 | 15068417 | 251 |
| 130 |  | 45787 | 19092306 | 416 | 51202 | 12784020 | 250 |
| 131 |  | 35810 | 14934472 | 417 | 49089 | 12627998 | 257 |
| 132 |  | 42117 | 17591174 | 418 | 56377 | 13963344 | 248 |
| 151 |  | 43623 | 18143786 | 416 | 68985 | 17224451 | 250 |
| 152 |  | 46066 | 19158324 | 416 | 56247 | 14331511 | 255 |
| 153 |  | 52073 | 21693519 | 417 | 68417 | 17671812 | 258 |
| 154 |  | 54934 | 22793666 | 415 | 73101 | 18670104 | 255 |
| 155 |  | 36282 | 15129032 | 417 | 58173 | 14699133 | 253 |
| 156 |  | 49911 | 20766300 | 416 | 68420 | 17221342 | 252 |
| Total |  | 4006740 | 1669331778 |  | 4969628 | 1259286659 |  |

Effective tags are the number of effective sequences of clean tags after filtering the chimera.

**Table S2** Bacterial and fungal biomarker species (at order level) in the bulk and rhizosphere soils

|  | Taxa | Groups | Means | LDA values | P values |
| --- | --- | --- | --- | --- | --- |
| Bacteria | *Actinobacteria* | RS | 5.12 | 4.06 | 0.01 |
| Fungi | *Capnodiales* | BS | 4.24 | 3.81 | <0.001 |
|  | *Dothideomycetes* | BS | 4.92 | 4.45 | <0.001 |
|  | *Hypocreales* | BS | 5.08 | 4.17 | 0.02 |
|  | *Helotiales* | BS | 4.54 | 4.00 | 0.01 |
|  | *Pleosporales* | BS | 4.78 | 4.31 | <0.001 |
|  | *Sordariales* | BS | 4.68 | 3.83 | 0.05 |
|  | *Sordariomycetes* | BS | 5.36 | 4.41 | 0.01 |
|  | *Leotiomycetes* | BS | 4.55 | 4.00 | 0.01 |
|  | *Cantharellales* | RS | 4.14 | 3.59 | 0.01 |

Means represents the logarithmic value of the average relative abundances of microorganisms in millions of times. LDA > 3.5 *p* < 0.05. The larger the LDA score value, the greater the impact of differential taxa.

**Table S3** Soil chemical properties in the bulk and rhizosphere soils

| Groups | TC  (g·kg^-1^) | TN  (g·kg^-1^) | TP  (g·kg^-1^) | TK  (g·kg^-1^) | NH_4_^+^-N  (mg·kg^-1^) | NO_3_^-^-N  (mg·kg^-1^) | AP  (mg·kg^-1^) | AK  (mg·kg^-1^) | pH |
| --- | --- | --- | --- | --- | --- | --- | --- | --- | --- |
| BS | 12.56±0.86 | 1.06±0.10 | 1.30±0.02 | 10.94±0.37 | 6.13±0.53 | 11.33±1.81 | 50.58±3.59 | 176.47±10.50 | 6.85±0.13 |
| RS | 16.32±1.46** | 1.56±0.14** | 2.19±0.26* | 12.25±0.52 | 8.27±0.51** | 11.65±2.35 | 107.87±20.06* | 201.95±10.18 | 6.81±0.11 |

Values in the table means ± standard errors (N=84). TC, TN, TP and TK, total contents of carbon, nitrogen, phosphorus and potassium, respectively; NH_4_^+^-N and NO_3_^-^-N, ammonium nitrogen and nitrate nitrogen; AP and AK, active contents of phosphorus and potassium, respectively. BS, bulk; RS, rhizosphere soil. ** (*p* < 0.01) and * (*p* < 0.05) mean significant differences between BS and RS.

**Table S4** Main properties of the correlation network of bacteria and fungi in the bulk and rhizosphere soils

| Network properties | Groups | | | | |
| --- | --- | --- | --- | --- | --- |
|  | Bacteria | |  | Fungi | |
|  | Bulk soil | Rhizosphere soil |  | Bulk soil | Rhizosphere soil |
| Total nodes | 140 | 98 |  | 69 | 91 |
| Total links | 2714 | 1895 |  | 108 | 215 |
| Positive links | 1356 | 947 |  | 108 | 211 |
| Negative links | 1358 | 948 |  | 0 | 4 |
| Average degree | 19.40 | 19.39 |  | 3.13 | 4.66 |
| Average clustering coefficient | 0.24 | 0.58 |  | 0.36 | 0.48 |
| Average neighborhood connectivity | 24.85 | 26.07 |  | 3.80 | 16.53 |
| Average edge Betweenness | 162.77 | 311.89 |  | 20.96 | 16.53 |
| Topological coefficient | 0.40 | 0.42 |  | 0.33 | 0.40 |

The correlation network analysis was performed based on the top 200 relative abundance of bacteria and fungi at OTU level; *p* < 0.05; The absolute values of correlation coefficient > 0.8.

**Table S5** Bacterial keystone species in the bulk and rhizosphere soils

| Bulk soil | | | | |  | Rhizosphere soil | | | | |
| --- | --- | --- | --- | --- | --- | --- | --- | --- | --- | --- |
| Node name | Degree | Clustering | Genus | Phylum |  | Node name | Degree | Clustering | Genus | Phylum |
| OTU8394 | 102 | 0.74 | *Acidothermus* | *Actinobacteriota* |  | OTU2193 | 104 | 0.67 | Unclassified *Elsterales* | Proteobacteria |
| OTU8294 | 101 | 0.70 | Unclassified *Roseiflexaceae* | *Chloroflexi* |  | OTU8394 | 102 | 0.66 | *Acidothermus* | *Actinobacteriota* |
| OTU12816 | 100 | 0.73 | Unclassified *Chloroflexi* | *Chloroflexi* |  | OTU2779 | 101 | 0.68 | Unclassified *Acidobacteriales* | Acidobacteriota |
| OTU6073 | 100 | 0.72 | Unclassified *Gammaproteobacteria* | *Proteobacteria* |  | OTU1607 | 100 | 0.70 | Unclassified *Acidobacteriales* | Acidobacteriota |
| OTU9347 | 100 | 0.76 | Unclassified *Elsterales* | *Proteobacteria* |  | OTU4129 | 99 | 0.71 | *Candidatus Solibacter* | *Acidobacteriota* |
| OTU4129 | 100 | 0.76 | *Candidatus Solibacter* | *Acidobacteriota* |  | OTU9989 | 99 | 0.70 | Unclassified *Gaiellales* | Actinobacteriota |
| OTU9646 | 100 | 0.76 | Unclassified *Gaiellales* | *Actinobacteriota* |  | OTU4126 | 99 | 0.71 | Unclassified *Chloroflexi* | *Chloroflexi* |
| OTU10450 | 99 | 0.77 | *Jatrophihabitans* | *Actinobacteriota* |  | OTU8453 | 98 | 0.72 | *Acidothermus* | *Actinobacteriota* |
| OTU9820 | 99 | 0.74 | Unclassified *Gaiellales* | *Actinobacteriota* |  | OTU9820 | 98 | 0.72 | Unclassified *Gaiellales* | Actinobacteriota |
| OTU2779 | 99 | 0.75 | Unclassified *Acidobacteriales* | *Acidobacteriota* |  | OTU10450 | 97 | 0.73 | *Jatrophihabitans* | *Actinobacteriota* |
| OTU8404 | 98 | 0.77 | *Sphingomonas* | *Proteobacteria* |  | OTU9646 | 97 | 0.73 | Unclassified *Gaiellales* | *Actinobacteriota* |
| OTU9988 | 98 | 0.76 | *Candidatus Udaeobacte* | *Verrucomicrobiota* |  | OTU7930 | 96 | 0.72 | Unclassified *Gaiellales* | *Actinobacteriota* |
| OTU3192 | 98 | 0.78 | Unclassified *Vicinamibacteria* | *Acidobacteriota* |  | OTU9347 | 96 | 0.71 | Unclassified *Elsterales* | *Proteobacteria* |
| OTU1607 | 98 | 0.77 | Unclassified *Acidobacteriales* | *Acidobacteriota* |  | OTU12825 | 96 | 0.72 | Unclassified *Nitrosomonadaceae* | *Proteobacteria* |
| OTU7631 | 98 | 0.76 | Unclassified *Elsterales* | *Proteobacteria* |  | OTU2 | 96 | 0.73 | *Candidatus Solibacter* | *Acidobacteriota* |
| OTU2431 | 97 | 0.75 | Unclassified *Chloroflexi* | *Chloroflexi* |  | OTU9244 | 96 | 0.69 | Unclassified *Micromonosporaceae* | Actinobacteriota |
| OTU2193 | 97 | 0.78 | Unclassified *Elsterales* | *Proteobacteria* |  | OTU3616 | 95 | 0.75 | *Phaselicystis* | *Myxococcota* |
| OTU6463 | 97 | 0.76 | Unclassified *Rokubacteriales* | *Methylomirabilota* |  | OTU11692 | 95 | 0.75 | *Ensifer* | *Proteobacteria* |
| OTU12825 | 97 | 0.76 | Unclassified *Nitrosomonadaceae* | *Proteobacteria* |  | OTU4172 | 94 | 0.74 | *Candidatus Solibacter* | *Acidobacteriota* |
| OTU2075 | 97 | 0.78 | Unclassified *Burkholderiales* | *Proteobacteria* |  | OTU3124 | 94 | 0.74 | *Haliangium* | *Myxococcota* |

Bacterial OTUs with the top 20 node degree (from highest to lowest) in the correlation network analysis are shown in the table.

**Table S6** Fungal keystone species in the bulk and rhizosphere soils

| Bulk soil | | | | |  | Rhizosphere soil | | | | |
| --- | --- | --- | --- | --- | --- | --- | --- | --- | --- | --- |
| Node name | Degree | Clustering | Genus |  |  | Node name | Degree | Clustering | Genus |  |
| OTU2991 | 37 | 0.45 | *Fusicolla* | *Ascomycota* |  | OTU3825 | 51 | 0.44 | *Pyrenochaetopsis* | *Ascomycota* |
| OTU2088 | 33 | 0.55 | *Cutaneotrichosporon* | *Basidiomycota* |  | OTU3414 | 47 | 0.49 | Unclassified *Pleosporales* | Ascomycota |
| OTU3040 | 33 | 0.51 | *Cystofilobasidium* | *Basidiomycota* |  | OTU5013 | 44 | 0.51 | *Sphaerosporella* | *Ascomycota* |
| OTU4047 | 32 | 0.42 | Unclassified fungi | Unclassified fungi |  | OTU5500 | 44 | 0.51 | *Sphaerosporella* | *Ascomycota* |
| OTU4750 | 32 | 0.40 | *Fusarium* | *Ascomycota* |  | OTU5150 | 43 | 0.55 | Unclassified *Rozellomycota* | *Rozellomycota* |
| OTU5598 | 31 | 0.42 | *Mortierella* | *Mortierellomycota* |  | OTU5598 | 43 | 0.48 | *Mortierella* | *Mortierellomycota* |
| OTU2694 | 30 | 0.61 | *Cystofilobasidium* | *Basidiomycota* |  | OTU2247 | 42 | 0.50 | Unclassified *Trechisporales* | *Basidiomycota* |
| OTU4418 | 30 | 0.38 | Unclassified *Chaetothyriaceae* | *Ascomycota* |  | OTU1391 | 40 | 0.60 | *Arthrographis* | *Ascomycota* |
| OTU2086 | 30 | 0.61 | *Arthropsis* | *Ascomycota* |  | OTU2088 | 39 | 0.62 | *Cutaneotrichosporon* | *Basidiomycota* |
| OTU1123 | 30 | 0.59 | *Tuber* | *Ascomycota* |  | OTU5599 | 39 | 0.56 | *Tomentella* | *Basidiomycota* |
| OTU76 | 29 | 0.46 | *Thielavia* | *Ascomycota* |  | OTU3051 | 38 | 0.65 | *Cladophialophora* | *Ascomycota* |
| OTU1399 | 29 | 0.42 | *Mortierella* | *Mortierellomycota* |  | OTU2473 | 38 | 0.43 | *Chaetomium* | *Ascomycota* |
| OTU3281 | 29 | 0.45 | *Fusicolla* | *Ascomycota* |  | OTU5498 | 38 | 0.55 | *Dichotomopilus* | *Ascomycota* |
| OTU3414 | 28 | 0.48 | Unclassified *Pleosporales* | *Ascomycota* |  | OTU3207 | 37 | 0.68 | *Clavulina* | *Basidiomycota* |
| OTU2033 | 28 | 0.59 | *Mortierella* | *Mortierellomycota* |  | OTU1014 | 37 | 0.68 | Unclassified *Ascomycota* | *Ascomycota* |
| OTU1391 | 27 | 0.64 | *Arthrographis* | *Ascomycota* |  | OTU5185 | 37 | 0.57 | *Mortierella* | *Mortierellomycota* |
| OTU5532 | 27 | 0.37 | *Cryptococcus* | *Basidiomycota* |  | OTU5546 | 36 | 0.50 | Unclassified *Biatriosporaceae* | Ascomycota |
| OTU3825 | 27 | 0.46 | *Pyrenochaetopsis* | *Ascomycota* |  | OTU2694 | 36 | 0.67 | *Cystofilobasidium* | *Basidiomycota* |
| OTU2583 | 27 | 0.64 | Unclassified *Hypocreales* | *Ascomycota* |  | OTU2325 | 36 | 0.64 | *Trechispora* | *Basidiomycota* |
| OTU5424 | 27 | 0.42 | Unclassified fungi | Unclassified fungi |  | OTU1123 | 36 | 0.69 | *Tuber* | Ascomycota |

Fungal OTUs with the top 20 node degree (from highest to lowest) in the correlation network analysis are shown in the table.

**Table S7** Bacteria functional abundances in the bulk and rhizosphere soils

| Function | BS | RS |
| --- | --- | --- |
| Chemoheterotrophy | 30.17±2.79 | 31.04±1.12 |
| Aerobic chemoheterotrophy | 28.35±3.42 | 29.53±1.40 |
| Animal parasite or symbionts | 4.32±1.53 | 3.85±0.62 |
| Human pathogens | 3.75±1.14 | 3.56±0.75 |
| Nitrate reduction | 3.74±1.65 | 3.12±1.12 |
| Human pathogens pneumonia | 3.26±1.02 | 3.21±0.79 |
| Nitrogen fixation | 2.49±1.44 | 2.70±1.27 |
| Cellulolysis | 2.20±1.70 | 2.60±1.79 |
| Aromatic compound degradation | 2.28±0.98 | 2.39±0.85 |
| Predatory or exoparasitic | 1.87±0.50 | 2.04±0.58 |

The top 10 functional abundance are shown in the table.
